# The Differentiation of Narrative Styles in Individuals with High Psychopathic Deviate

**DOI:** 10.1007/s10936-021-09824-w

**Published:** 2021-12-06

**Authors:** Barbara Gawda

**Affiliations:** Department of Psychology of Emotion and Personality, Maria Curie-Skłodowska University, Lublin, Poland

**Keywords:** Psychopathic deviate traits, Linguistic material, discourse styles, Narratives, Assessment

## Abstract

The current study was designed to show the differentiation of narrative styles in individuals with high scores in Psychopathic deviate (Pd) scale and develop a method enabling identification of psychopathic personality traits based on linguistic indicators. 600 spontaneous narrations related to emotional topics have been examined for grammar, syntactic, and lexical indicators. The indicators have been selected based on a review related to language of psychopaths. The narrations were written by 200 persons who were also tested for psychopathic deviate and intelligence level, including prisoners diagnosed with antisocial personality disorder. Independent judges identified the linguistic indicators which were then counted for each person with the use of computer software. The configuration profiles of the linguistic indicators/narrative styles were established using k-mean clustering method. Then, ANOVA was performed to show which clusters differentiate the levels of psychopathic deviate. The findings show there are two configurations of language features (important: single features were not examined) associated with high levels of psychopathic deviate patterns. Two narrative styles were identified, labelled *demonstrative-digressive-egocentric-emotional-dogmatic* and *reserved-focused on the topic-repetitive*, which indicate high psychopathic deviate traits. The ROC curves were applied to establish the prediction of the narrative styles for high psychopathic deviate scores.

## Introduction

Language of psychopaths has been studied for many years. It has been assumed that their language is a rich source of information on their dysfunctional emotionality and personality. Since Cleckley (1988), psychopaths have frequently been described as displaying *semantic dementia*; it is believed that their speech is empty as they use “words without meaning” and are blind to the meaning of words. Studies focusing on the processing of emotional and neutral words support the opinion that psychopaths are less capable in semantic processing of emotional words (Williamson, 1991; Williamson et al., 1991). They have deficits in making use of emotional polarity of words, which suggests problems in recognizing their emotional valence (Williamson, 1991). Although there are many opinions concerning prisoners’ language, for example, that their language attributes are dependent on the needs of psychopathic offenders, i.e., they use more emotional and seductive words to arouse the listener’s attention (Sneiderman, 2006), research related to this topic is ‘fledgling’, as stated by Gullhaugen and Sakshaug (2019). One of the problems related to generalizations of results on psychopaths’ language is linked to the fact that the related studies have mainly been carried out in prisons. It has been taken into consideration that individuals confined to a prison setting score lower in phonological processing as well as reading and writing activities (Brites et al., 2015), and therefore the findings related to the language of psychopaths should not be generalized. In order to synthesize the existing evidence connected with this issue, the following sections review the findings on the general impressions related to the speech of psychopaths, lexical aspects, syntactic aspects, grammar and differences in focus in their communications.

### General Impressions Related to Speech

There are different opinions on the ways psychopaths communicate. It has been highlighted that those with high emotional intelligence are able to express feelings in more convincing ways, especially when they aim to deceive others (Porter et al., 2011). They are perceived as highly verbal and able to tell impressive self-serving stories (Cleckley, 1988). Psychopathy positively correlates with talkativeness and dominance (Manson et al., 2014; Rimé et al., 1978). Psychopaths tend to excessively use jargon and poorly integrated phrases; they also have troubles adhering to one train of thought (Gillstrom & Hare, 1988). Psychopaths’ language is persuasive, fluid, and misleading (Hare, 1993; Reimer, 2008). Furthermore, some inconsistency in the quality of voice expressing affective and neutral words has been documented. When psychopaths produce affective words, a so-called split off effect (they speak more quietly, treat affective words as devoid of emotion, they seem to be oblivious to the affective valence of the speech) can be observed for a shorter or longer duration. This was interpreted as an indicator of affect isolation by the defense mechanism (Louth et al., 1998).

### Lexical Aspects

With regard to lexical aspects of psychopathic speech, many different tendencies have been observed. Psychopaths tend to use categorical words such as *never, nothing*, or *always* more frequently, which is interpreted to reflect their mental rigidity, rigid worldview, and a tendency towards generalization (Gawda, 2013). The use of categorical words is thought to be related to rigid thinking (“black and white thinking”) which can be also present in other personality and mental disorders (Johnson, 2010). Another feature of their speech is that they use contradictory words and sentences; the level of self-contradictions is much greater in this group (Adshead, 2014; Helfgott, 2004). They also manifest withdrawal tendency which is shown by words or phrases that contradict previous expressions (Rieber & Vetter, 1994). Such contradictions in their speech can be manifested with the use of phrases or words conveying aversion without a reason (Adshead 2014), and tendency to omit important story aspects, which gives an impression of inconsistent speech (Porter & Woodworth, 2007). Other research on lexical aspects of their speech showed that they use more negations in their speech, such as *not, no, nothing, never* (Gawda, 2010; Helfgott, 2004; Rieber & Vetter, 1994). Furthermore, it has been observed that they use more nouns (Hancock et al., 2011), and fewer academic references i.e. few specialized terms (Sneiderman, 2006). The first can be linked to lower speech dynamics, while the second one to lower richness of their vocabulary. Notably, their emotional language is overflowing with lexical inconsistencies. Some studies showed that while psychopaths speak about emotional events, they are less emotionally expressive, in particular, they produce anxiety related words less frequently and use less emotionally intense language (Brinke et al., 2017; Hancock et al., 2011; Hildebrand et al., 2004; Le et al., 2017). In contrary to these findings, psychopaths have been found to be more expressive in talking about anxiety, love, and hate (Gawda, 2010, 2013). However, some of emotional descriptions of higher intensity words were not related to the specific stimulus (Gawda, 2013). It has been shown that sometimes they use emotional words without being aware of its emotional valence and meaning. Their speech may give an impression of being expressive, however, the adequacy of used words is not very appropriate.

### Syntax

In the context of syntax analysis focusing on psychopathic offenders’ speech, it has been found that they use past tenses more frequently (Hancock et al., 2011), as well as repetitions, pauses, and negations in affective speech (Gawda, 2010). Some researchers point out to the lack of coherence in the psychopaths’ speech, also in terms of logical reasoning as well as poor flow, stuttering, or rapid shifts in topic (Adshead, 2014; Brinkley et al., 1999; Hancock et al., 2011; Holmqvist, 2008; Le et al., 2017; Selenius & Strand, 2015; Williamson et al., 1991). It has been also observed that psychopathic offenders come to conclusions based on insufficient information (Gawda, 2013).

### Grammar

Psychopaths do not respect grammatical rules in their speech (Eichler, 1965). They produce ambiguous texts and introduce contradictive information in their speech (Hare, 1993, 2003). Brites et al. (2015) found that psychopaths know phonological, syntactic, morphological, and semantic rules of verbal and written language. They recognize word structure and its principles, as well as phonetic and morphological features (Brites, 2016). Brites (2016) concludes that psychopaths’ language has an adaptive value and reflects the main characteristics of their personality.

### Structure of Discourse

It has been found that narratives constructed by psychopathic offenders are less structured and lack temporal perspective (Keltikangas-Jarvinene, 1982). Psychopaths do not describe emotional context, or they can focus on negative aspects of the situation (Dolan & Anderson, 2002). Analysis of their contents showed that the texts contain more negations, qualifiers (*suppose, more* or *less*), retractions (words or phrases partly detracting from the preceding statement), and evaluations. Hence, their texts are less clear (Eichler, 1965). However, different types of coherence and cohesion are connected to psychopathy in varied ways (Williamson, 1991). This association also depends on the type of produced texts. Lexical and conjunctive cohesion has been found to be negatively associated with psychopathy. On the other hand, referential cohesion and incomplete references have not been found to be associated with this disorder. Importantly, this finding relates to neutral stories but not to affective narratives. Coherence measured by plot-unit analysis was also investigated in psychopaths. Williamson (1991) found that neutral stories told by psychopaths were poorer in cohesion than their affective stories; fewer plot-units were produced by psychopathic subjects in affective stories. Psychopaths do not include in their stories all the information needed. Their speech reflects a failure to link actions and resolutions within their narratives. Notably, lack of cohesion in texts produced by psychopaths is not always reported, and depends on some factors, most importantly on the anxiety level. It was concluded that only psychopaths experiencing greater anxiety produced less coherent texts (Brinkley et al., 1999).

### Situational Variations

Some studies suggest that properties of psychopaths’ speech or their way of expression depend on the topic/situation. These situational factors include control or spontaneous communication, also referred to as implicit and explicit communication. Romberg et al. (2015) examined three psychopaths (with video recording) While psychopathic offenders were perceived to be controlled, their speech was monotonous and less emotional. When psychopaths were emotionally activated, their speech contained numerous disturbances, breaks, inappropriate laughing, and reflected difficulties in articulation. The study suggests that the explicit verbal expression is defensive, while implicit expression presents contradictory style, featuring inappropriate sounds, atypical melody, excessive breaks, or rapid speech (Romberg et al., 2015). This shows that psychopath’s speech varies depending on the topic, emotional activation, and perceived control (Adshead, 2004; Frodi et al., 2001; Le et al., 2017; Romberg et al., 2015; Weiss et al., 2016). It could mean that this way of speaking reflects psychopathic offenders’ tendency to reduce negative tension (Romberg et al., 2015) and avoid negative feelings (Adshead, 2014; Le et al., 2017). The findings on the implicit/explicite differentiation of the psychopaths’ speech reflect their difficulties in emotional regulation.

### Hypotheses

Given the above data on many potential patterns of language associated with psychopathy, the present study aimed at creating a holistic method to enable indication of psychopathic personality traits based on linguistic indicators. We intent to show the differentiation of discourse in individuals with high Pd (psychopathic deviate) traits. Importantly, the study was not intended to explain how single specific linguistic properties are related to psychopathic deviate traits. The purpose was instead to establish whether grouped patterns of narrative indicators/narrative styles related to psychopathic deviate traits exist or not. We assume that there are groups of linguistic markers/narrative styles which could allow to differentiate individuals with high and low psychopathic deviate traits. Additionally, we attempt to establish predictive values of the identified narrative styles in searching any configuration of linguistic characteristics being a basis for the ‘prediction-focused‘ research on psychopathic personality traits in the future.

## Method

### Participants and Procedure

The study involved a sample of 200 male subjects, aged between 18 and 50 years, right-handed, heterosexual. This sample included three groups: prisoners diagnosed with antisocial personality disorder by clinicians and displayed high scores in Psychopathic deviate scale (*n* = 60), prisoners without antisocial personality disorder and with low scores in Psychopathic deviate scale (*n* = 40), non-prisoners without antisocial personality disorder and with low scores in Psychopathic deviate scale (*n* = 100). To assess psychopathic personality traits, the Minnesota Multiple Personality Inventory 2nd edition revised version (Polish adaptation) was used (Butcher et al., 2012). The three groups were established based on cut-score according to the instruction in the MMPI-2: the group with high score has scores higher than 74 T, the groups with low T scores have scores lower than 45 (Butcher et al., 2012). The prisoners were convicted for multiple serious crimes against health (offences involving grievous bodily harm), life, and public order. They did not present other neuropsychiatric and somatic disorders. These groups were matched in terms of intelligence, education, and other characteristics such as age, lack of other psychiatric, neurological, or somatic disorders (demographic and other data based on the screening questionnaire). The three groups differed in level of Psychopathic deviate traits (Table 1). The three groups were selected as they do not differ in verbal intelligence, education level, neurological problems, neuropsychiatric history in order to assess psychopathic facets effect on linguistic production. The experimental protocol was approved by the Local Ethics Committee of the Maria Curie-Sklodowska University in Lublin, Poland. The permission for the study from the involved prisons has been obtained. The control group was selected from the adult male population in different regions in Poland. They were recruited through advertisement in the university. The subjects voluntarily participated in the examination; they were not paid for their participation. They all were of Polish origin. After the procedure was explained to the subjects, they provided written informed consent to participate in the study. The examination of prisoners took place in five state prisons in the psychological offices, while examination of the control group at schools or at the University; they performed the tasks in calm rooms.

**Table 1:** Comparisons between the groups

Variables	Prisoners with high Pd n = 60 *M (SD)*	Prisoners with low Pd n = 40 *M (SD)*	Non-prisoners with low Pd n = 100 *(M, SD)*	*F*_*(2, 197)*_
Age	35.5 (11.00)	34.2 (10.00)	33.5 (9.80)	1.45
Education	10.27 (1.80)	10.40 (1.50)	10.44 (1.50)	0.66
Vocabulary	43.95 (8.00)	45.01 (7.00)	45.15 (7.00)	1.04
Pd	80.98 (5.03)	44.94 (10.44)	30.73 (2.14)	232.77***
Length PN	35.90 (6.68)	19.65 (5.04)	18.64 (2.32)	14.04***
Length NN-H	33.29 (4.40)	18.75 (3.31)	18.74 (9.03)	14.16***
Length NN-A	28.18 (6.78)	16.81 (3.74)	15.87 (3.14)	16.56***

*M* mean, *SD* standard deviation, *Pd* Psychopathic deviate scales T scores, *PN* length of the positive stories, *NN-H* length of the negative stories about hate, *NN-A* length of the negative stories about anxiety

***p < 0.001

After a screening procedure, the participants completed the subtest Vocabulary from the WAIS-R, the MMPI-2-®, and narrative tasks. Each person wrote three texts (one about love, one about hate, and one about a situation involving anxiety). The subjects were asked to write a story inspired by photographs showing emotional situations (photo elicitation method). They received the instruction: “Look at the picture. Imagine that you are one of the people in the photograph. Write a story about it.” Intelligence was tested because it is known to be one of the key factors determining speech parameters, as well as written and spoken performance.

### Measures

- Vocabulary subtest from the WAIS-R: The Wechsler Adult Intelligence Scale-Revised is a general test of intelligence, based on 15 subtests. The WAIS-R can measure four index scores and the full-scale IQ. Vocabulary scores were taken into consideration in the present study to compare the participants’ verbal skills because creation of a narrative depends on verbal intelligence (Brzeziński et al., 2004).^1^
- The Minnesota Multiphasic Personality Inventory 2nd edition—revised form in Polish adaptation by Brzezinska and colleagues (MMPI-2-®). The MMPI-2-® is a self-report personality assessment tool that contains 567 true/false test items (Butcher et al., 2012). The MMPI-2’s psychometric properties are appropriate. The reliability of the Pd scale (with K correction) is 0.79 (from Polish adaption by Brzezinska and others). Cronbach’s alpha for Pd scale (without K correction) is 0.69 (Butcher et al., 2012). In the current study, Cronbach’s alpha for Pd scale is 0.792. With regards to the MMPI–2-® validity, it has been found that this measure “appears to hold promise in the assessment of both affective-interpersonal and social deviance” (Sellbom et al., 2005, p. 341). Although Pd scale does not describe psychopathy comprehensively, it allows to assess psychopathic traits, especially antisocial lifestyle traits such as impulsiveness, lack of realistic long-term goals, irresponsibility, and antisocial behavior. It has been found that MMPI-2-® significantly correlates with other psychopathy measures (Graham, 2012), including non-questionnaire measures. In particular, it significantly correlates with antisocial behavior measured by the PCL-R (Hansen et al., 2013). The Pd raw scores were transformed into T scores and multiplied by 0.4 K scales scores. The T scores of the Pd scale were used in the statistical analyses here. The Pd scale is devoted to measure antisocial behavior, problems with respecting social rules and law, impulsivity, lack of reflectivity, lack of responsibility, and hostile behavior (Butcher et al., 2012). The aim was to establish the relationship between some facets of psychopathic personality and groups of linguistic indicators.
- Narrative discourse about emotional situations. The participants wrote in total 600 narratives, 200 about positive emotional situations, 200 about quarrel/hate, and 200 about anxiety. All these narrations were analyzed by three independent psychologists, who identified linguistic indicators. The indicators were selected taking into account all the data presented in the meta-analyses referred to in the Introduction. The indicators were counted with the use of computer software designed to count words based on an Excel formula. First, all self-references, negations, categorical words, etc. were entered in the database. Then, a mathematical formula was applied to count all the words representing specified linguistic categories. Next, a narrative produced by a subject was uploaded into the software and numerical scores for all the types of linguistic categories were programmed. Finally, all the scores were checked by the psychologists.

The following linguistic indicators were assessed and counted in the stories:

- Positive situation (love): number of emotional words, number of nouns, number of verbs, number of words off-topic (e.g. sadness), number of pauses (defined here as a crossed-out letter, a crossed-out word and unwanted sentence amended later), number of repetitions (e.g., “*I am very, very happy*…”), number of negations (e.g. “*I am not sure that.*.”), number of references to self (the total for: verbs in the first person singular [e.g., *I feel, I want*: Brown and Heimberg (2001) argued that it refers to self-concentration/egocentrism which could be the indices of narcissism], pronouns and other parts of speech referring to the first person singular [e.g., *my, me*, etc.]), number of categorical words/expressions (e.g. *never, nothing, each, every, all*);
- Negative situation (hate): number of emotion words, number of nouns, number of verbs, number of words off-topic (e.g. *happiness*), number of pauses, number of repetitions, number of negations (e.g. “*I am not sure that*…”), number of references to self (1st person singular references), number of categorical words/expressions (e.g. *never, each, every, all, nothing*);
- Anxiety situation: number of emotion words, number of nouns, number of verbs, number of words off-topic (e.g. *happiness*), number of pauses, number of repetitions, number of negations (e.g. “*I am not sure that*…”), number of references to self (1st person singular references), number of categorical words/expressions (e.g. *never, every, each, all, nothing*);
- Number of evaluations (e.g. *on the whole, in general all women*…);
- Length of a story measured by number of words.

### Statistical Analyses

Three groups were compared in terms of intelligence, psychopathic deviate, and demographic characteristics using one-way analysis of variance. Then, k-mean clustering was used to establish profiles of linguistic indicators. This technique allows to identify groups of persons who present similar styles of narrative, i.e. similar discourse types. These groups form clusters. Distances between final cluster centers were calculated. To show whether the final cluster centers differentiate psychopathic deviate traits, ANOVA and *post hoc* (Scheffe test) comparisons were performed. In the end, the analysis of ROC curves was performed to test predictive values of the final clusters which significantly differentiated persons with high level of psychopathic deviate from those with low psychopathic deviate.

## Results

One-way Anova showed that the three groups did not differ from each other in the level of verbal intelligence, age, nor education level. They significantly differed in Psychopathic deviate level (Pd scores); prisoners with high levels of Psychopathic deviate traits, prisoners with low Psychopathic deviate scores and non-prisoners with low level of Psychopathic deviate traits (Table 1). The findings show that the differences between the groups in the linguistic characteristics are not due to verbal nor linguistic capacities but are related to personality or emotional patterns.

**Fig. 1:**
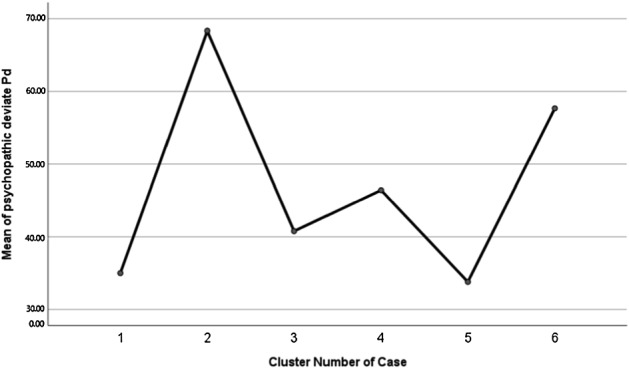
Means plot: final cluster centers in relation to Psychopathic deviate

To demonstrate synthetic linguistic characteristics of the psychopathic individuals, a k-mean clustering analysis was performed. It revealed six independent final cluster centers, i.e., six different discourse styles (Table 2). The distances between them are shown in Table 3. Each final cluster center differs from the other centers and presents a configuration of linguistic properties of written discourse (Table 2). To show whether these final cluster centers differentiate Psychopathic deviate scores, Anova was performed, which showed significant differences between the final clusters (*F*_(6, 193)_ = 7.09, *p *< 0.001) and *post hoc* comparisons (Table 4). Two final cluster centers/profiles among the six centers, encompassing linguistic indicators, are associated with high levels of Psychopathic deviate scores. These are final cluster centers number 2 and 6. These results are illustrated in Fig. 1. The final cluster center no. 2 encompasses the following main linguistic characteristics: long narrative; many emotional words used in emotionally positive and negative situations except for those involving anxiety; highly frequent use of emotional phrases unrelated to the situation; fewer nouns than verbs in descriptions; very frequent use of self-references; large number of categorical words, negations, and evaluating phrases in various situations. This type of speech may be described as *demonstrative-digressive-egocentric-emotional-dogmatic*. The final cluster center no 6 encompasses the following main linguistic characteristics: average number of words, i.e., average length of the narrative; similar number of emotional words in positive and negative situations; similar number of nouns and verbs; numerous repetitions in positive and negative situations; few words in a situation involving anxiety; infrequent words unrelated to the topic. This type of speech may be referred to as *reserved-focused on the topic-repetitive*. There are also three final cluster centers/three profiles related to low level of Psychopathic deviate scores (multiple comparisons between final cluster centers are presented in Table 4). These are final cluster centers number 1, 3 and 5 (see Fig. 1).

**Table 2:** Final cluster centers

Linguistic indicators	Cluster					
	1	2	3	4	5	6
	n = 12	n = 13	n = 50	n = 14	n = 85	n = 26
P. emotion words	11.50	17.33Φ	6.98	10.79	3.54	11.83
P. nouns	3.50	5.33Φ	1.72	2.43	1.02	4.17Φ
P. verbs	1.50	8.00Φ	2.32	4.21	0.83	4.00Φ
P. off-topic	0.00	4.00Φ	0.22	1.29	0.12	1.00Φ
P. pauses	0.50	2.33Φ	1.00	1.14	0.37	1.00
P. repetitions	2.00	6.00	2.72	5.36	0.59	7.00Φ
P. negations	0.00	3.67Φ	0.48	0.79	0.14	1.17
P. self	1.50	10.67Φ	3.16	7.29	1.42	7.67
P. categorical	1.00	1.33Φ	0.48	1.14	0.24	1.33Φ
N. emotion words	23.00	13.67Φ	6.52	7.36	3.46	9.50
N. nouns	6.50	5.33	2.44	2.36	1.07	4.17
N. verbs	10.50	6.00	2.46	3.21	1.49	3.33
N. off-topic	0.00	1.00Φ	0.50	0.57	0.14	0.50Φ
N. pauses	1.50	1.33	0.74	1.00	0.62	1.00
N. repetitions	7.00	2.67	1.94	1.86	0.48	1.67
N. negations	4.50	4.33Φ	1.36	2.00	0.72	3.67
N. self	10.50	10.67Φ	2.56	4.00	1.26	4.00
N. categorical	2.00	1.33	0.74	1.57	0.30	1.50
A: emotion words	10.00	6.33	6.52	8.29	3.02	6.67
A. nouns	3.00	1.00Φ	1.86	2.36	0.80	1.00Φ
A. verbs	6.00	2.33	2.68	3.21	0.98	2.67
A. off-topic	1.00	1.67	0.62	0.43	0.22	1.67
A. pauses	0.50	1.00	0.86	0.79	0.27	1.33
A. repetitions	4.00	3.00	2.16	2.57	0.42	4.00Φ
A. negations	1.50	3.00Φ	1.40	1.50	0.75	1.67
A. self	6.00	6.00Φ	3.20	5.50	1.42	5.33
A. categorical	1.50	1.67Φ	0.56	0.86	0.23	1.17
Evaluation	4.00	3.67Φ	1.30	1.79	1.04	1.83
Story length	30.00	115.33Φ	31.92	57.71	32.45	87.33Φ

*P*. positive situation, *N*. negative situation, *A*. anxiety situation

Φ*p*<0.001

**Table 3:** Distances between final cluster centers

Final cluster centers	1	2	3	4	5	6
1		87.43	22.93	34.95	33.71	60.63
2	87.43		85.77	59.44	106.14	30.55
3	22.93	85.77		26.76	20.91	56.18
4	34.95	59.44	26.76		47.36	30.02
5	33.71	106.14	20.91	47.36		76.53
6	60.63	30.55	56.18	30.02	76.53

**Table 4:** Multiple comparisons of Pd scores between final cluster centers

Scheffe test						
(I) Cluster number of case	(J) Cluster number of case	Mean difference (I–J)	*SE*	*p*	95% Confidence interval	
					Lower bound	Upper bound
1	2	− 35.83	14.23	0.28	− 83.69	12.02
	3	− 8.28	11.24	0.99	− 46.08	29.52
	4	− 13.92	11.78	0.92	− 53.56	25.70
	5	− 1.29	11.11	1.00	− 38.66	36.07
	6	−24.33	12.73	0.60	− 67.14	18.47
2	1	35.83	14.23	0.28	− 12.02	83.69
	3	27.55	9.26	0.12	− 3.61	58.71
	4	21.90	9.92	0.43	− 11.45	55.26
	5	34.54	9.11	0.01**	3.90	65.17
	6	11.50	11.02	0.95	− 25.57	48.57
3	1	8.28	11.24	0.99	− 29.52	46.08
	2	− 27.55	9.26	0.12	− 58.71	3.61
	4	− 5.64	4.71	0.92	− 21.50	10.20
	5	6.98	2.60	0.21	− 1.78	15.76
	6	− 16.05	6.73	0.34	− 38.70	6.59
4	1	13.92	11.78	0.92	− 25.70	53.56
	2	− 21.90	9.92	0.43	− 55.26	11.45
	3	5.64	4.71	0.92	− 10.20	21.50
	5	12.63	4.39	0.14	− 2.13	27.41
	6	− 10.40	7.60	0.86	− 35.98	15.17
5	1	1.29	11.11	1.00	− 36.07	38.66
	2	− 34.54	9.11	0.01**	− 65.17	− 3.90
	3	− 6.98	2.60	0.21	− 15.76	1.78
	4	− 12.63	4.39	0.14	− 27.41	2.13
	6	− 23.04	6.51	0.03*	− 44.95	− 1.12
6	1	24.33	12.73	0.60	− 18.47	67.14
	2	− 11.50	11.02	0.95	− 48.57	25.57
	3	16.05	6.73	0.34	− 6.59	38.70
	4	10.40	7.60	0.86	− 15.17	35.98
	5	23.04	6.51	0.03*	1.12	44.95

*SE* standard error

**p < 0.01, *p < 0.05

**Fig. 2:**
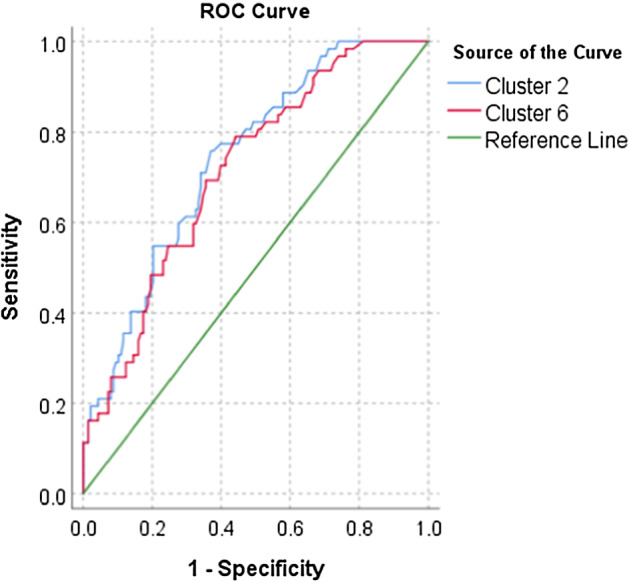
The ROC curves for Cluster no 2 and no 6: sensitivity, specificity

**Fig. 3:**
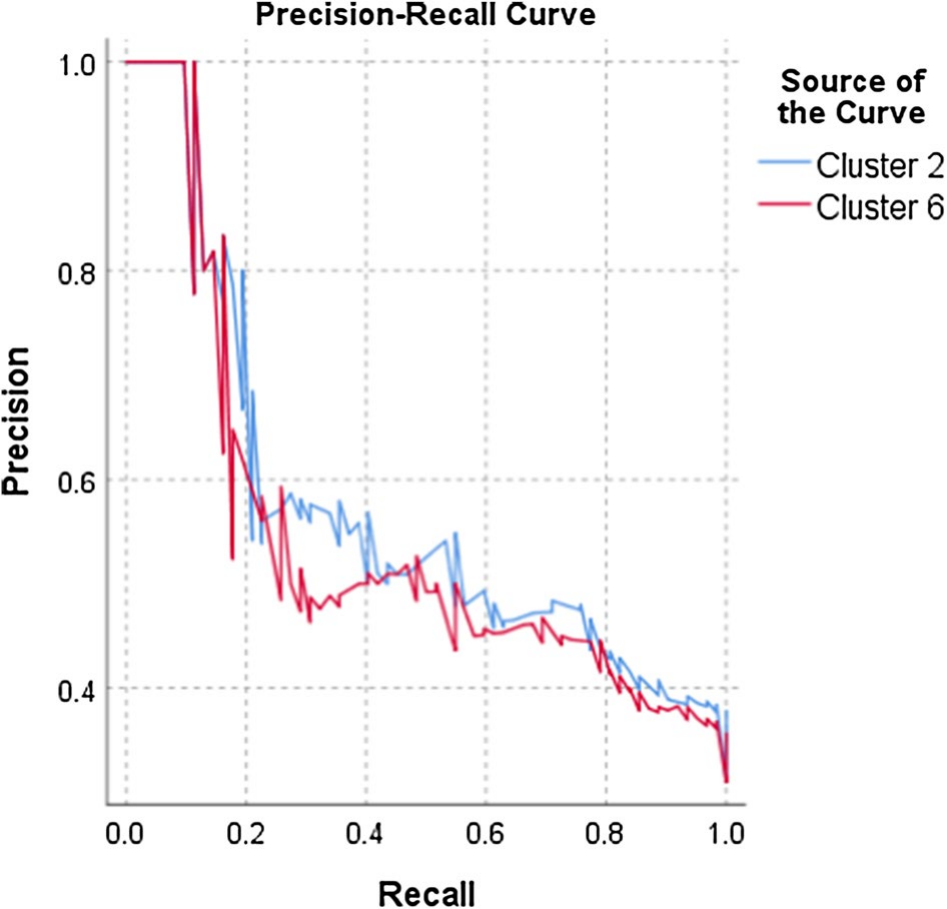
The ROC curves for Cluster no 2 and no 6: precision

**Fig. 4:**
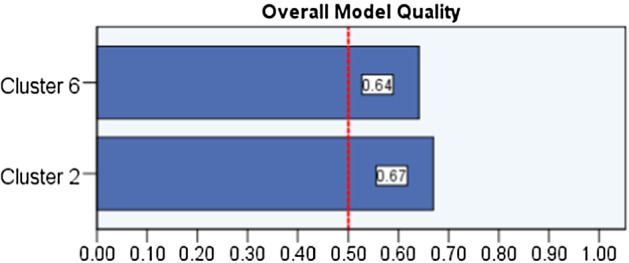
The ROC curves: overall model quality

To test the predictive values of the final cluster no 2 and no 6 in terms of indication of high Psychopathic deviate scores, the ROC curves analyses were performed. First, the two variables were created—Cluster 2 and Cluster 6—as they were found to be the most associated with the Pd scale. These two clusters contained the appropriate linguistic variables found during the k-mean clustering analysis. For the Cluster 2 and 6, the ROC curves analyses were conducted to show their values in prediction of high Pd scores (Figs. 2 and 3). The obtained models for Cluster 2 and 6 are of good quality (Fig. 4). The ROC curves analyses showed that Cluster 2 and 6 demonstrate high sensitivity,0.758, and 0.790, respectively (basing on the cutoff in Table 5). These analyses revealed that specificity of these measures is average; Cluster 2 = 0.377, Cluster 6 = 0.435. Precision of these clusters have been found as well, their values are Cluster 2 = 0.466, Cluster 6 = 0.444. The Gini index is average (Table 5). In sum, the ROC curves analyses showed that Cluster 2 and 6 have some predictive properties for identifying high Psychopathic deviate. It means that the sensitivity of Cluster 2 and 6 in terms of indication for Pd is appropriate (Fig. 2) while specificity and precision are average (Fig. 3).

**Table 5:** The ROC curves analysis

Variables	AUR	SE	*p*	CFI 95%		Gini Index	Cutoff
				Lower bound	Upper bound		
Cluster 2	0.740	0.036	0.000	0.670	0.810	0.479	74.5
Cluster 6	0.714	0.037	0.000	0.641	0.787	0.427	81.5

*AUR* area under the ROC curve, *SE* standard error

## Discussion

Studies focusing on the language of psychopaths show the number of characteristics which differentiate their linguistic expression from that of non-psychopathic subjects. Based on the related literature, a number of potential language features was selected and taken into account in our study. This study was designed not to determine whether these single features differentiate subjects with high level and low level of Psychopathic deviate, but to investigate to what extent complexes/configurations of language features are different in these individuals and whether or not this configuration enables prediction of psychopathic deviate patterns. Given the fact that profiling based on isolated features is of little value, the current analysis took into account configurations of language features. Analyses based on the method of k-means clustering allowed to identify that there are two profiles/complexes of language features which are significantly associated with high level Psychopathic deviate. These profiles are varied in terms of lexical, syntactic, and grammatical features as well as discourse structure. The profiles are referred to as: *demonstrative-digressive-egocentric-emotional-dogmatic* and *reserved-focused on the topic-repetitive.* It documents that there are different narrative styles in persons with high Psychopathic deviate. These linguistic profiles suggest that they are significantly related to typical symptoms of psychopathic personality. The former profile, i.e., *demonstrative-digressive-egocentric-emotional-dogmatic*, reflects several traits reported in the related literature. Individuals meeting this profile tend to speak a lot, use more emotional phrases and often seductive words to attract attention (Sneiderman, 2006), they are convincing, especially when their aim is to deceive others (Porter et al., 2011). At the same time their narratives frequently deviate from the main topic, contain a lot of digressions, and negative statements and focus on the speaker. This profile and linguistic expression are consistent with opinions that psychopaths can make a good impression, are perceived as highly verbal, talkative, and able to tell evocative self-serving stories (Cleckley, 1988; Manson et al., 2014; Rimé et al., 1978). Elaborate tale concentrating on the psychopathic person’s self, emotional, containing many negative and dogmatic (categorical) phrases, and frequently deviating from the main theme, is a consequence of many emotional difficulties experienced by such individuals. Generally, these individuals display deficits in recognizing emotional valence of words (Williamson, 1991), and present tendencies towards high dominance, mental rigidity, and egocentrism (Manson et al., 2014; Rimé et al., 1978). This results in the use of overelaborated vocabulary and at the same poor integration of linguistic expression, which reflects difficulties in adhering to one train of thought (Gillstrom & Hare, 1988). This *demonstrative-digressive-egocentric-emotional-dogmatic* style of talking is emphasized in the literature by statements that psychopaths’ speech is persuasive, fluid, and misleading (Hare, 1993; Reimer, 2008). Some inconsistencies in the syntactic and lexical aspects of speech produced by psychopathic individuals are linked to their defense mechanisms and withdrawal tendency (Louth et al., 1998; Rieber and Wetter, 1994). Lack of coherence in the speech of psychopaths in terms of logical reasoning as well as poor flow, stuttering or rapid shifts in topic, reported by many researchers (Adshead, 2014; Brinkley et al. 1999; Hancock et al., 2011; Holmqvist, 2008; Le et al., 2017; Selenius & Strand, 2015; Williamson et al., 1991), is consistent with the *demonstrative-digressive-egocentric-emotional-dogmatic* style of talking shown in the present study. Potentially, that is why they use contradictive words and sentences (Adshead, 2014; Helfgott, 2004), categorical terms (dogmatic words) such as *never, nothing*, or *always* more frequently, and usually evaluate or generalize. Profile no 2, described above and labelled *demonstrative-digressive-egocentric-emotional-dogmatic*, also reflects their rigid worldview and lack of mental flexibility. The frequent use of negation is also potentially related to negative/egocentric worldview (Gawda, 2010). It has been found that psychopaths tend to use more phrases or words conveying aversion, for no apparent reason (Adshead, 2014; Porter & Woodworth, 2007), and more commonly use negation in their speech, such as *not, no, nothing, never* (Gawda, 2010; Helfgott 2004; Rieber & Vetter, 1994). The interesting tendency for more frequent use of nouns has not been confirmed in this study. Possibly, this fact is observed while comparing psychopaths and non-psychopaths, but not when constructing profiles of configuration of linguistic indicators associated with psychopathic personality (Hancock et al., 2011). While speaking about emotional situations psychopaths use fewer nouns than verbs in both positive and negative narratives. Another interesting aspect of this profile is that those individuals know a lot of emotional vocabulary (with the exception of situations involving anxiety), which is contradictory to opinions suggesting that they are less emotionally expressive and produce less emotionally intense language while speaking about emotional events (Brinke et al., 2017; Hancock et al., 2011; Hildebrand et al., 2004; Le et al., 2017). The *demonstrative-digressive-egocentric-emotional-dogmatic* style of speech presented by individuals with high Psychopathic deviate shows that they could be more expressive in talking about emotionally positive and negative situations and at the same time less focused on the topic (Gawda, 2010, 2013). This profile can be associated with elevated level of anxiety as it was found that psychopathic individuals with high anxiety produce less coherent speech (Brinkley et al., 1999).

The next profile/cluster no 6 identified in the present study, i.e., *reserved-focused on the topic-repetitive*, illustrates individual differences in group with Psychopathic deviate. This type of individuals produces shorter narratives, not so convincing, related to the topic, and at the same time containing a lot of repeated words. This may mean that their speech is not fluent, however, it is not digressive. An interesting tendency has also been observed in this style, namely that the number of nouns and verbs used in this type of narratives is similar. Since the use of verbs is thought to be associated with mental dynamics, the latter observation may be associated with the fact that these individuals present poorer mental dynamics. Another aspect of this profile is that the use of vocabulary related to situations involving anxiety is rather poor. This is consistent with other findings showing that psychopathic individuals less frequently produce words related to anxiety (Hancock et al., 2011), and more commonly use repetitions and pauses in affective narratives (Gawda, 2010). The use of repetitions is associated with a lower number of words in general and lower number of emotional words. When an appropriate term cannot be found in one’s personal lexicon, some repetitions are introduced in order to fill the gap. This is related to the more frequent use of negations, qualifiers (*suppose, more* or *less*), or retractions (words or phrases partly detracting from the preceding statement), and lower level of fluency in the texts (Eichler, 1965). Although the utterance is focused on the relevant topic the person has problems finding appropriate phrases and words.

To conclude, the language of individuals with Psychopathic deviate is varied. The differentiation of talking style is potentially linked to the type of psychopathy (Mokros et al., 2015), situational factors, topic, emotional activation, and interactions between these aspects as well as other personality traits. With regard to psychopathy types, numerous propositions have been formulated, and three types of psychopaths have been identified: manipulative, aggressive, and sociopaths (Mokros et al., 2015). It seems plausible that differentiation of language of persons with Psychopathic deviate is associated with these types. Notably, the relationship between language and personality is not simple, it is potentially mediated or moderated by other factors.

The second aim of this research was to establish the predictive properties of the two main linguistic profiles associated with high Psychopathic deviate scores. The appropriate sensitivity of profiles named *demonstrative-digressive-egocentric-emotional-dogmatic* and *reserved-focused on the topic-repetitive* in terms of indication Psychopathic deviate was confirmed. Other predictive values of these two linguistic profiles are found as average, i.e., their specificity and precision are not sufficient. It suggests that two main styles of narration named *demonstrative*… and *reserved*… are sensitive measures of Psychopathic deviate, however, further research is needed to establish other predictive validity properties.

## Conclusions

Research into psychopathic individuals’ discursive expression is of importance because the findings allow to describe main emotional and personality deficits associated with this condition and enable better understanding of emotional and other dysfunctions of those affected. The language of persons with Psychopathic deviate is differentiated which means there are varied styles of communication. The two profiles of linguistic characteristics identified in the study and labelled: *demonstrative-digressive-egocentric-emotional-dogmatic* and *reserved-focused on the topic-repetitive*, show there is a configuration of linguistic properties associated with high level of psychopathic deviate. These two linguistic styles/profiles partly allow to predict psychopathic deviate/antisocial behavior based on the linguistic material. High sensitivity of these two profiles was established. These results are promising for predictive validity in terms of prediction personality pathology on linguistic patterns.

This finding can be informative for forensic linguistic profiling. It can help to construct a description of the offender’s personality or/and demographic characteristics. Forensic psycholinguistics is focused on the examination of relationships between psychological features, e.g. impulsivity, anxiety, depression, paranoia (Grieve et al., 2011). Such comprehensive examination allows to identify language features associated with some personality traits (Kredens & Roszkowski, 2007). On the whole, examinations of discourse offer information useful in determining offenders’ identities, truthfulness, personality characteristics, and even their potential for violence (Grant, 2010; Oxburgh et al., 2010).

### Limitations

The research presented here has some limitations. First, a target sample included only male prisoners. Although the population of prisoners is important in terms of prevalence of psychopathic personality, research on non-prisoner psychopathic sample is needed to understand their language. There is also a need to include women sample with psychopathic personality, both incarcerated and non-incarcerated women. It would be also of value to increase the sample size. Geographic origin is potentially a limitation too; all participants were of Polish origin and the research was conducted in Polish language. It is possible that there are differences in linguistic characteristics between English- and Polish-speaking psychopathic persons. Although scientists point out that there are more similarities between Polish and English emotional words than, for instance, between English and Japanese (e.g. Wierzbicka, 1992), it seems plausible that some linguistic patterns associated with psychopathy manifest differently in these two languages. To our knowledge, there is no research on these dissimilarities. It could be an interesting topic for future research: the relationship between psychopathy and language moderated by geographic origin. Another important limitation is the tool we used; the MMPI-2® is not a comprehensive measure of psychopathy. It allows to assess antisocial behavior and some psychopathic facets such as hostility, impulsivity, irresponsibility, and problems with reflectivity. The MMPI-2® was used because the presented research is a part of larger program focused on the searching relationship between psychopathology and language. However, in terms of psychopathy prediction, the MMPI-2® Pd scale has limitations.

### Future directions

The presented way of analyzing speech and predicting psychopathic traits, based on the configuration of linguistic markers, needs future verifications, particularly in terms of other type of spontaneous narratives produced by different persons with different types of disorders. The examination focused on searching predictive validity is needed to establish appropriate precision and specificity of the linguistic profiles associated with psychopathic traits.

## Footnotes

1 There is no significant correlation between Vocabulary scores and Pd scores (r = − 0.061 ns), neither between Vocabulary and final clusters (V-Cramer = 0.065 ns).
